# Effect of child marriage on girls' school dropout in Nepal: Analysis of data from the Multiple Indicator Cluster Survey 2014

**DOI:** 10.1371/journal.pone.0180176

**Published:** 2017-07-20

**Authors:** Kazutaka Sekine, Marian Ellen Hodgkin

**Affiliations:** 1 Health Section, UNICEF Nepal, Kathmandu, Nepal; 2 Education Section, UNICEF Nepal, Kathmandu, Nepal; International Center for Research on Women, UNITED STATES

## Abstract

School dropout and child marriage are interrelated outcomes that have an enormous impact on adolescent girls. However, the literature reveals gaps in the empirical evidence on the link between child marriage and the dropout of girls from school. This study identifies the ‘tipping point’ school grades in Nepal when the risk of dropout due to marriage is highest, measures the effect of child marriage on girls’ school dropout rates, and assesses associated risk factors. Weighted percentages were calculated to examine the grades at highest risk and the distribution of reasons for discontinuing school. Using the Nepal Multiple Indicator Cluster Survey (MICS) 2014 data, we estimated the effect of marriage on school attendance and dropout among girls aged 15–17 by constructing logistic regression models. A multivariate logistic regression model was used to assess risk factors of school dropout due to child marriage. It was found that early marriage is the most common reason given for leaving school. Overall, the risk of school dropout due to marriage heightens after girls complete the fifth or sixth grade. The risk of girls’ dropping out peaks in the seventh and eighth grades and remains noteworthy in the ninth and tenth grades. Married girls in Nepal are 10 times more likely to drop out than their unmarried peers. Little or no education of the household head, belonging to the Kirat religion, and membership of a traditionally disadvantaged social class each elevate the risk of school dropout due to early marriage. The findings underscore the need to delay girl’s marriage so as to reduce girls’ school dropout in Nepal. School-based programmes aimed at preventing child marriage should target girls from the fifth grade because they are at increased risk of dropping out, as well as prioritizing girls from disadvantaged groups.

## Introduction

School dropout and child marriage are interrelated outcomes that have an enormous impact on adolescent girls, curtailing full realization of their rights, limiting their livelihood options, and harming their health and wellbeing as well as that of their children. There are also broader social implications related to economic development and gender equality. Education is a fundamental human right for all children, as enshrined in the Convention on the Rights of the Child (1989), and is seen as an enabling right essential for the fulfilment of other rights thanks to the empowering impact of education on societies and individuals [1]. As such, education was prioritized as part of the Millennium Development Goals (MDGs) and the subsequent Sustainable Development Goals (SDGs). SDG 4 focuses on the right to education for all children, expanding the vision beyond equal access, to encompass issues relating to equity and quality [2].

Nearly 58 million children of primary school age (typically between 6 and 11 years) around the world were not enrolled in school in 2012 [3]. A disproportionate number of these out-of-school children of primary school age were girls; 10% of all girls and 8% of all boys were denied the right to education [4]. In addition, 63 million children of lower-secondary school age (typically between 12–15 years) were out of school worldwide in 2012 [3]. South Asia has the second largest population of primary and lower-secondary school-aged children who are out of school in the world [4]. While it is estimated that the number of primary-aged children who are out of school fell by 42 million between 2000 and 2012 globally, the Millennium Development Goal of achieving universal primary education by 2015 was not achieved [4]. Sustainable Development Goal 4 recognizes that this gap must be closed. Goal 4 aims to ensure inclusive and equitable quality education and promote lifelong opportunities for all [2]. Addressing persistent inequities relating to gender, economic and geographic differences is a key component of SDG 4. The analysis of statistical trends relating to education and the practice of child marriage linked to socioeconomic disparities will inform work towards these global targets and highlight potential areas of focus for practitioners and policy makers.

While boys often face economic pressures to drop out of school related to migration and labour, child marriage is globally considered a major barrier to retaining girls in school. South Asia has some of the highest rates of child marriage in the world, with more than one third of all child marriages that occur globally occurring in the region [5]. Nepal, alongside Bangladesh, Afghanistan, and India, is considered a ‘hot-spot’ country in South Asia, as the practice of child marriage is pervasive [5].

It is argued that education is a powerful positive predictor of female age at marriage [6–10]. Girls with no education are three times more likely to marry or enter into union before age 18 than those with a secondary or higher education [5]. Schooling defers girls’ age at marriage, especially if they attend secondary education [11]. Once they are no longer in school, however, girls are more likely to be viewed as marriageable [12], which leads to a heightened vulnerability to early marriage. Further, married girls are drastically less likely to attend school than their unmarried peers [13]. A study examining data from 27 sub-Saharan African countries found that each additional year of early marriage reduces the girl’s individual probability of having some secondary school education by 5.6 percentage points, and it reduces the girl’s individual probability of completing secondary school by 3.5 percentage points [14]. In Bangladesh, each additional year of delay in the female age at marriage had the effect of increasing schooling by 0.22 of a year and literacy rate by 5.6 percentage points [15].

However, association does not equate to causation. Although the strength of association is one of the Bradford Hill criteria used to make an overall judgement about a causal relationship [16], data drawn from cross-sectional surveys do not indicate causality [7]. Experts continue to debate the direction of influence. Are girls pulled from school to be married or are they married at a young age because they drop out of school or are pulled out of school? The causality between child marriage and girls’ school dropout may go both ways. Child marriage deprives girls of educational opportunities. However, supply side issues such as poor access to school, low quality of educational provision, and poor skilled employment prospects, as well as demand side challenges such as concerns over safety and security or the need for unpaid household labour may be important contributing factors to child marriage. Girls may therefore discontinue their education due to a range of factors and then, as they are unable to complete their education, enter into marriage.

Decisions on these life events are complex, multifaceted, and most often private family decisions. As Nguyen [14] argues in the African context, the educational prospects of a girl are likely to contribute to the decisions made by her (or her parents) regarding whether to stay in school or get married, which means that decisions about the timing of leaving school and early marriage are often made concomitantly. This makes it technically difficult to elucidate the causal order of girl’s early marriage and school dropout. Although the link between child marriage and school dropout of girls deserves greater attention, there is little empirical evidence available on this issue. What is largely unknown is the grade or age at which the risk of school dropout due to marriage escalates as well as the factors that exacerbate that risk. Therefore, while education is believed to be ‘the single most powerful antidote to early marriage’ [7], more research is needed to better understand how policies and interventions can better target those girls at risk of dropping out of school or marrying early. This study is intended to contribute to the literature by measuring the effect of child marriage on girls’ school attendance and dropout as well as identifying risk factors associated with school dropout due to child marriage.

This paper assesses the effect of child marriage on school dropout among Nepali girls. The four important questions that this study sought to answer are as follows: (i) How common is school dropout due to marriage among girls? (ii) What is the ‘tipping point’ grade at which the risk of leaving school due to marriage escalates? (iii) How much more likely (or less likely) is school attendance and school dropout among married girls compared to their unmarried peers? (iv) Who is more likely to leave school due to marriage?

## Materials and methods

### Data

Data used in this study were extracted from the Nepal Multiple Indicator Cluster Survey (MICS) 2014 [17]. The cross-sectional survey is one of the largest household surveys conducted in Nepal, and it was designed to provide up-to-date data for monitoring the situation of children and women. In the survey, a nationally representative sample was obtained through use of a two-stage, stratified random sample design. In the first stage, urban and rural areas within each region were identified as the main sampling strata. Within each stratum, a specific number of census enumeration areas were selected based on probability proportional to size with oversampling of urban areas. In the second phase, a household listing was carried out within the selected enumeration areas, and then a systematic sample of 25 households was drawn in each sample enumeration area. These procedures resulted in a sample of 14,162 women of reproductive age (15–49 years) and a response rate of 95%.

To address our multiple research questions, we used two subsamples of the MICS data for analyses. First, analysis of the prevalence of school dropout due to child marriage and analysis of the grade of school in which girls are at the highest risk of leaving due to marriage were both limited to women aged 20–24. We chose this age range because women are either no longer enrolled or have completed secondary school by age 20. Second, to examine the effect of child marriage on school attendance and school dropout, as well as to assess risk factors associated with school dropout due to marriage, we restricted analyses to girls aged 15–17. Focus on older adolescents is appropriate, as the large majority of those married as minors in Nepal are in this age range. Furthermore, the MICS data do not include respondents below the age of 15.

### Measures

This study began by looking at the reason for discontinuing education using descriptive statistics. The reason for school dropout was a categorical variable which was assessed by the question, ‘What was the main reason you did not continue your studies further?’ The possible reasons provided in the questionnaire were ‘economic reasons’, ‘parents did not allow’, ‘got married’, ‘school facility far away’, ‘need to do household work’, ‘did not like to study’, ‘physical disability’, and ‘other’. We also analysed the highest grade completed by women who left school due to child marriage. The highest grade was treated as an interval variable in the analysis.

We examined three dependent variables of interest, using regression analyses. Firstly, the school attendance variable dichotomized whether girls were attending school or not when the survey was conducted. This variable was coded as ‘1’ if a girl was attending school, ‘0’ otherwise. Second, the school dropout variable also dichotomized whether girls withdrew from school before completing secondary education (the twelfth grade, or approximately 17 years old, in the context of Nepal). This variable was coded as ‘1’ if a girl was out of school, ‘0’ otherwise. Grade repetition was not taken into account in the variable. These two variables were assessed using the question, ‘Are you currently enrolled in any school?’

Another dependent variable differentiated between unmarried girls attending school and married girls who left school due to marriage. Married girls who left school and reported marriage as the main reason for leaving school were coded as ‘1’, and girls who were unmarried and attending school at the time of the survey were coded as ‘0’. Girls who left school for other reasons were dropped from this variable.

Child marriage was used as the independent variable in regression analyses on school participation and school dropout. A dichotomous variable was made for the variable based on reported age at first marriage. In this study, a girl was considered married if she was formally married or informally in union before the age of 18.

The covariate variables used in the multivariate logistic regression models were demographic and socioeconomic characteristics which were assessed by questions within the MICS questionnaire. The covariate variables include:

- age
- place of residence (urban vs. rural)
- household wealth status
- religion
- social class
- the level of education of the household head.

A relative index of household wealth, which represents economic status, was calculated based on a standard set of interviewer-observed assets, including the ownership of consumer items and dwelling characteristics. Household wealth status was determined by a wealth score given to each household in the total sample based on the assets owned by that household; women were then divided into poor (lower 40%) and non-poor (upper 60%). Religion was classed as Hindu, Buddhist, Muslim, Kirat, Christian, and others. Social class as defined by caste was dichotomised as Dalit or non-Dalit based on ethnicity data. The education level of the household head was classed according to the highest level of education attended ranging from no education to primary education, and secondary or higher education.

### Data analysis

Data analyses in this study comprise descriptive analyses and logistic regression analyses. Weighted percentages were calculated to examine the distribution of the reasons for leaving school, and the highest grade of school dropout due to child marriage. Similarly, weighted percentages were calculated to compare demographic and socioeconomic characteristics among married and unmarried girls. Pearson’s chi-squared test was used for categorical variables to determine whether differences in demographic and socioeconomic characteristics were statistically significant between married and unmarried girls. Student’s t-test was used for continuous variables for the same purpose. We considered a two-tailed p-value of < 0.05 to be statistically significant.

The likelihood of school attendance and school dropout for married girls was assessed by constructing logistic regression models and estimating unadjusted odds ratios for the dependent variables of school participation and school dropout. We regressed each dependent variable against child marriage. Further, adjusted odds ratios were estimated after controlling for demographic and socioeconomic characteristics (age, place of residence, household wealth status, religion, social class, and education of the household head). Prior to calculating adjusted odds ratios, Pearson's correlation coefficients were run to test possible collinearity among these covariates.

By constructing a multivariate logistic regression model, the likelihood of unmarried girls attending school or married girls having left school due to child marriage was assessed to identify the predictor of school dropout due to child marriage. We regressed the girl’s status against age, place of residence, household wealth status, religion, social class, and education of the household head, which were treated as independent variables in this model.

All the data were weighted to account for the complex sample design, such as stratified sampling and probabilities of unequal sample selection between regions. Using the national women’s weighting for the entire MICS allows for analyses that produce nationally representative estimates. All statistical analyses for this study were conducted using Stata 13 (StataCorp LP, College Station, TX).

### Ethics statement

The Nepal MICS procedures were reviewed and approved by the Nepal Central Bureau of Statistics and UNICEF. Because this study involves secondary data analyses of a publicly available dataset which does not reveal the identity of the respondents, ethical approval from respective institutions was not required.

## Results

### The prevalence of leaving school due to child marriage

Table 1 shows that among girls aged 20–24, marriage was cited as the leading reason for discontinuing school in 40% of cases.

**Table 1:** Reasons for school dropout among women aged 20–24 (n = 1092).

Reason	%
Got married	39.8
Did not like to study	21.7
Economic reasons	15.3
Need to do household work	14.9
Parents did not allow	5.7
School facility far away	2.0
Physical disability	0.7

Data are given in weighted percentages.

### Risk of leaving school due to child marriage: The tipping point grades

Table 2 shows the distribution of the highest grade completed by women aged 20–24 who left school due to child marriage. Median grades of dropout were the seventh and eighth grades, accounting for 42.8% together. Between 11–14% of women dropped out of school due to marriage after completing the fifth, sixth, ninth or tenth grade.

**Table 2:** Highest grade completed among women aged 20–24 citing marriage as the main reason for dropping out of school (n = 326).

Grade	%
1	0.0
2	0.3
3	1.6
4	5.1
5	11.4
6	11.1
7	21.0
8	21.8
9	13.8
10	13.8

Data are given in weighted percentages.

### The likelihood of school attendance and school dropout for married girls

The Nepal MICS data generated a subsample of 1,631 girls aged 15–17. Participants reported a mean age of 16.0 years (SD = 0.79). Descriptive statistics for the demographic and socioeconomic characteristics of the girls included in this study are presented in Table 3. Most girls (88.2%) were unmarried. Among those who were married, 31.2% were attending school, and 68.8% were out of school. Among those who were unmarried, 84.9% were attending school, and 15.1% were out of school. More than 80% of the participants attended secondary school. The population that was represented was predominantly rural, non-Dalit, and Hindu. Nearly half of the girls’ household heads had not attended primary school.

**Table 3:** Child marriage prevalence as it correlates with demographic and socioeconomic characteristics of girls aged 15–17 in Nepal.

		Participants (n = 1,631)		Married (n = 202)		Unmarried (n = 1,429)		P-value for difference in covariates by marital status
Married								
	Yes	202	(11.8%)	-	-	-	-	
	No	1,429	(88.2%)	-	-	-	-	
Schooling ^a^								P < 0.001
	In-school	1,093	(78.5%)	53	(31.2%)	1,040	(84.9%)	
	Out-of-school	258	(21.5%)	111	(68.8%)	147	(15.1%)	
Highest level of education								P < 0.001
	None	73	(5.8%)	23	(15.0%)	50	(4.6%)	
	Primary	180	(11.0%)	38	(20.2%)	142	(9.8%)	
	Secondary	1,178	(69.2%)	127	(60.4%)	1,051	(70.4%)	
	Higher	200	(14.0%)	14	(4.3%)	186	(15.2%)	
Current age								P < 0.001
	15	520	(30.9%)	22	(13.9%)	498	(33.2%)	
	16	613	(39.0%)	68	(32.9%)	545	(39.8%)	
	17	498	(30.2%)	112	(53.2%)	386	(27.1%)	
Place of residence								P = 0.004
	Urban	307	(14.2%)	30	(7.7%)	277	(15.1%)	
	Rural	1,324	(85.8%)	172	(92.3%)	1,152	(84.9%)	
Ecological zone								P = 0.806
	Mountain	449	(7.0%)	53	(6.2%)	396	(7.2%)	
	Hill	606	(42.6%)	84	(43.9%)	522	(42.5%)	
	Terai	576	(50.3%)	65	(50.0%)	511	(50.4%)	
Household wealth status								P = 0.072
	Poor	885	(42.7%)	127	(50.1%)	758	(41.7%)	
	Non-poor	746	(57.3%)	75	(49.9%)	671	(58.3%)	
Religion								P = 0.058
	Hindu	1,332	(83.7%)	164	(81.1%)	1,168	(84.0%)	
	Buddhist	155	(7.00%)	12	(4.7%)	143	(7.3%)	
	Muslim	41	(4.1%)	8	(7.9%)	33	(3.6%)	
	Kirat	65	(2.8%)	16	(4.9%)	49	(2.5%)	
	Christian	34	(2.3%)	2	(1.4%)	32	(2.4%)	
	Others	4	(0.2%)	0	(0%)	4	(0.2%)	
Social class ^b^								P < 0.001
	Non-Dalit	1,437	(88.5%)	157	(76.9%)	1,280	(90.1%)	
	Dalit	192	(11.5%)	45	(23.1%)	147	(10.0%)	
Education of household head ^c^								P < 0.001
	None	738	(45.1%)	113	(60.3%)	625	(43.0%)	
	Primary	341	(21.5%)	51	(24.5%)	290	(21.1%)	
	Secondary or higher	545	(33.3%)	38	(15.2%)	507	(35.8%)	

Data are n (weighted %).

Absolute number of participants does not perfectly correspond to percentages presented because weighted analyses were used.

^a^ n = 1,351

^b^ n = 1,629

^c^ n = 1,624

There were significant differences in the demographic and socioeconomic characteristics between girls aged 15–17 who were married and unmarried. Women who were married at a young age were more likely to have dropped out of school, have no formal education, reside in a rural setting, live in poverty, or have an uneducated household head, and they tended to be older adolescents, Dalit, or Muslim.

Table 4 presents the schooling and marital status of girls aged 15–17. The cross-tabulation shows that 77.0% of the respondents were attending school and unmarried. Nearly one out of ten girls (8.2%) were married and out of school. A small percentage of girls (3.9%) were married but stayed in school.

**Table 4:** Number and percentage of girls aged 15–17 by schooling status and marital status (n = 1,351).

	Married		Unmarried	
In school	53	(3.9)	1,040	(77.0)
Out of school	111	(8.2)	147	(10.9)

Data are n (weighted %).

Table 5 and S1 Table present odds ratios and 95% confidence intervals (CI) for school attendance among girls aged 15–17. The logistic regression analysis shows that married girls were significantly less likely to attend school (OR 0.08; 95% CI 0.05–0.13) compared to their unmarried peers. The effect remained significant (adjusted odds ratio (AOR) = 0.10; 95% CI 0.06–0.17) after adjusting for demographic and socioeconomic variables (age, place of residence, household wealth status, religion, social class, and education of the household head).

**Table 5:** Associations between child marriage and school participation.

	OR	95% CI	Adjusted OR^a^	95% CI
Married	0.08***	[0.05, 0.13]	0.10***	[0.06, 0.17]

n = 1351 for OR; n = 1344 for adjusted OR. OR = odds ratio; CI = confidence interval

^a^ Analysis adjusted for age, place of residence, household wealth status, religion, social class, and education of the household head.

*** Level of significance *p* < 0.001

The logistic regression analysis also shows that married girls were significantly more likely to leave school (OR 12.40; 95% CI 7.54–20.40) compared to their unmarried peers (Table 6 and S2 Table). The effect remained significant (AOR = 10.04; 95% CI 5.84–17.25) after adjusting for the same demographic and socioeconomic variables.

**Table 6:** Associations between child marriage and school dropout.

	Unadjusted OR	95% CI	Adjusted OR^a^	95% CI
Married	12.40***	[7.54, 20.40]	10.04***	[5.84, 17.25]

n = 1351 for OR; n = 1344 for adjusted OR. OR = odds ratio; CI = confidence interval

^a^ Analysis adjusted for age, place of residence, household wealth status, religion, social class, and education of the household head.

*** Level of significance *p* < 0.001

### Risk factors associated with school drop-out due to child marriage

Multivariate logistic regression analysis for the status of girls aged 15–17 (unmarried and attending school vs. having left school due to marriage) reveals that the age of girls, the level of education of the household head, and religion were the main contributing factors to school dropout due to child marriage (Table 7). As their age increased, girls were more likely to have dropped out of school due to marriage (AOR = 2.81; 95% CI 1.73–4.56). Girls living in a household with a household head with no education (AOR = 4.44, 95% CI 1.57–12.56) or who had only attended primary education (AOR = 4.56, 95% CI 1.65–12.61) were significantly associated with school dropout due to child marriage, compared to their peers living with a household head who attended secondary or higher school. Girls who identify as belonging to the Kirat religion were more likely to drop out of school due to child marriage (AOR = 2.31, 95% CI 1.00–5.34). While not strongly associated, social class as defined by caste was linked with school dropout due to child marriage at a significance level of 0.08 (AOR 2.24, 95% CI 0.92–5.43). Place of residence and household wealth status were not associated with school dropout due to early marriage. An unexpectedly low AOR was found for girls from rural areas or poor households.

**Table 7:** Influence of socioeconomic factors on the status of girls aged 15–17 (unmarried and attending school vs. having left school due to child marriage) (n = 1,042).

Variable		Adjusted ORs	95% CI
Age^a^		2.81*	[1.73, 4.56]
Place of residence^b^			
	Rural	Reference	
	Urban	1.18	[0.48, 2.92]
Household wealth status^c^			
	Non-poor	Reference	
	Poor	0.95	[0.52, 1.76]
Religion^d^			
	Hindu	Reference	
	Buddhist	0.77	[0.14, 4.39]
	Muslim	-	-
	Kirat	2.31*	[1.00, 5.34]
	Christian	0.46	[0.07, 2.97]
	Others	-	-
Social class^e^			
	Non-Dalit	Reference	
	Dalit	2.24	[0.92, 5.43]
Education of the household head^f^			
	Secondary or higher	Reference	
	Primary	4.56*	[1.65, 12.61]
	No education	4.44*	[1.57, 12.56]

Note. OR = odds ratio; CI = confidence interval

^a^ Analysis adjusted for place of residence, household wealth status, religion, social class, and education of the household head

^b^ Analysis adjusted for age, household wealth status, religion, social class, and education of the household head

^c^ Analysis adjusted for age, place of residence, religion, social class, and education of the household head

^d^ Analysis adjusted for age, place of residence, household wealth status, social class, and education of the household head

^e^ Analysis adjusted for age, place of residence, household wealth status, religion, and education of the household head

^f^ Analysis adjusted for age, place of residence, household wealth status, religion, and social class

* Level of significance *p* < 0.05

## Discussion

The current study shows that marriage was the most common reason given for girls’ school dropout. Overall, the risk of school dropout due to marriage heightens after girls complete the fifth or sixth grade. The risk of girls dropping out peaks in the seventh and eighth grades and remains high in the ninth and tenth grades. The analysis also demonstrates that married girls aged 15–17 in Nepal are 10 times more likely to leave school than are their unmarried peers. Little or no education of the household head, belonging to the Kirat religion or historically disadvantaged social class elevate the risk of school dropout due to child marriage. Girls living in a household with a household head who had not attended school or who had only attended primary school are approximately 4.5 times more likely to leave school due to child marriage, compared to having a household head who attended secondary school or higher. Girls belonging to the Kirat religion are 2.3 times more likely to drop out of school due to child marriage than their Hindu peers. Dalit girls are 2.2 times more likely to leave school due to child marriage, compared to their peers belonging to other castes.

The very high percentage (40%) of Nepali women reporting marriage as the main reason for school dropout is exceptional. The percentage of women aged 20–24 in sub-Saharan African countries who reported early marriage as the main reason ranged from 2% in Togo, Benin, and Côte d'Ivoire to 28% in Chad [18]. Since, in Nepal, marriage is more socially acceptable than other possible reasons, it may have been more frequently reported as the primary reason for leaving school, even if other factors are more attributable. Decisions about marriage and dropout are the result of a complex interplay of various factors. Marriage could be potentially used as an ex post justification for leaving school rather than being the actual cause. Girls choosing to cite a positive social status (marriage) rather than less desirable factors (poverty or lack of academic interest) may have influenced the results. However, this in itself indicates the social dimensions of the issue of child marriage and the need to address social norms around marriage and the aspirations of girls.

These findings on the significant effect of child marriage on girls’ education is consistent with those of earlier studies [13, 15, 18], suggesting that marriage curtails girls’ schooling. The incompatibility between marriage and schooling is partly attributed to social norms towards married girls in Nepal. Because of safety and security concerns, especially fear of rape and abduction, restrictions on married girls’ movements by their husbands and parents-in-law are common particularly in rural areas where many villages have no secondary school and girls are forced to travel outside their village [19]. Social pressure to protect the family’s honour could lead to families keeping married girls at home, which results in absenteeism and then dropout [20]. Marital status may also reinforce the family perception that they are no longer children who require formal education or the notion that that the returns on investment in education are negligible [21]. Despite relatively low costs in the form of fees, books, and transport, as well as fairly good access to schools in much of the country, marriage puts an end to girls’ education.

The risk of school dropout due to early marriage is exacerbated among girls living in a household with a household head with little or no formal education, from historically disadvantaged social class, and belonging to a certain religion. The finding that the level of education of the household head has a significant influence on the probability of school dropout due to child marriage demonstrates the inter-generational impact of educational attainment. Furthermore, as close to 30% of household heads in Nepal are women [17], retaining girls in school has the potential to not only curtail child marriage rates in this generation, but in generations to come. However, this study suggests that poverty is not a factor that further heightens the risk of school dropout due to child marriage. This finding is contrary to our expectations, as feeding, clothing and educating daughters is considered an economic burden on poor families [20, 22]. Moreover, families are motivated to marry off their daughters at a young age because dowry requirements often increase with the age of the bride [23, 24]. The finding implies that conditional cash transfers, which have been found by systematic reviews to have a positive impact on reducing child marriage in other contexts [12, 25], may not be effective in reducing child marriage in Nepal.

Evidence generated from this study can aid policy makers and education planners by helping them better understand the magnitude of the effect of child marriage on school attendance, who is at the highest risk of school dropout due to child marriage, and in which grades they face the highest risk of dropping out. The evidence could potentially inform policy making and advocacy for eliminating child marriage. Nepal has pledged to end child marriage by 2030 [26], but needs to accelerate its progress towards this goal. The findings guide better targeting of programmes to end child marriage and education programmes aimed at improving school retention. Formal education programmes need to support girls as they transition between grades, particularly with a focus on secondary education and those in the fifth to tenth grades. Since child marriage practices are deeply rooted in gender and social norms in a patriarchal society, it is also important to work with males relatives (fathers, fathers-in-low and husbands) of girls at risk of early marriage, as well as male school teachers. Further, non-formal education programmes need to be scaled up to reach the large proportion of girls who marry and drop out of school, in order to mitigate the harmful effects that early marriage can have and to halt the inter-generational impact of school dropout and child marriage.

The present study has several limitations that should be considered. First, the MICS data are prone to recall and social desirability biases because of their use of self-reported data. Possibly, the illegality of child marriage may have led to systematic under-reporting because of fear of legal repercussions. If underreporting of child marriage was the case, the study may have underestimated the effect of child marriage. In Bangladesh, however, a study found widespread misreporting of age of marriage, with more than half of women reporting their age at first marriage as younger than it actually was [27]. The MICS data collection team attempted to collect accurate data from interviewed women by ensuring privacy and confidentiality. Female interviewers administered a questionnaire in an attempt to increase the respondents’ comfort level.

Second, the cross-sectional nature of the MICS data does not permit the investigation of sequential events and causality. Age at the time of leaving school was not gathered in the Nepal MICS, so it was not possible to establish whether or not school leaving preceded marriage. Given the strength of the association and the frequency of reporting marriage as the main reason for school dropout, however, it is reasonable to argue that child marriage is a key factor that leads girls to leave school. Third, the household wealth index was based on assets of the current household that girls live in. For married girls, household wealth assessed in the survey did not consider the parental household. The potential inconsistency of economic status may have introduced an unsystematic bias in the analyses. Similarly, the education data of the household head was based on the household that married girls were living in at the time of the survey. Fourth, the analyses of the risk of school attendance and school dropout were limited to girls aged 15–17, and therefore the findings are not generalizable to other age groups in the country.

The strength of this study is that it deals with nationally representative data which was generated by a well-established national survey that used standardized measures and protocols. On the other hand, using panel data that track girls over time is a more credible way of establishing sequential events and causality between school dropout and child marriage. While not addressed in this study, further work to understand the impact of child marriage on boys who marry early is also needed.

## Conclusion

The current study yields solid evidence that child marriage significantly increases girls’ risk of school dropout in Nepal. Despite the limitations discussed above, the strength of the association and the frequency of reporting of marriage as the main reason for school dropout are remarkable enough to warrant the conclusion that child marriage is a main driver of girls’ dropping out of school in Nepal. This finding underscores the need to delay marriage in order to reduce gender disparity against females in school retention and the out-of-school population in many parts of the country. Reducing the risk of dropout requires strategies that retain girls in school and facilitate a smooth transition to secondary education. School-based programmes aimed at preventing child marriage should target girls from the fifth grade because of their escalated risk, and they need to prioritize girls from disadvantaged groups.

## Supporting information

S1 Table Associations between child marriage and school participation. (DOCX)

S2 Table Associations between child marriage and school dropout. (DOCX)

## References

[1] United Nations. Convention on the Rights of the Child. 1989. Available from: http://www.ohchr.org/EN/ProfessionalInterest/Pages/CRC.aspx.

[2] United Nations Economic and Social Council. Progress towards the Sustainable Development Goals: Report of the Secretary-General. 2016. Available from: http://www.un.org/ga/search/view_doc.asp?symbol=E/2016/75&Lang=E.

[3] UNESCO Institute for Statistics (UIS). Progress in getting all children to school stalls but some countries show the way forward. 2014. Available from: http://unesdoc.unesco.org/images/0022/002281/228184E.pdf.

[4] UNESCO Institute for Statistics (UIS), UNICEF. Fixing the broken promise of education for all: findings from the global initiative on out-of-school children. 2015. Available from: http://www.uis.unesco.org/Education/Documents/oosci-global-exsum-en.pdf.

[5] UNFPA. Marrying too young; End child marriage. 2012. Available from: http://www.unfpa.org/sites/default/files/pub-pdf/MarryingTooYoung.pdf.

[6] Sah N. How useful are the demographic surveys in explaining the determinants of early marriage of girls in the terai of Nepal. Journal of Population Research. 2008;25(2):207–22.

[7] Brown G. Out of wedlock, into school: combating child marriage through education. 2012. Available from: http://educationenvoy.org/wp-content/uploads/2013/09/Child-Marriage.pdf.

[8] Aryal TR. Age at first marriage in Nepal: differentials and determinants. J Biosoc Sci. 2007;39(5):693–706. doi: 10.1017/S0021932006001775 . 17156587 10.1017/S0021932006001775

[9] Glick P, Handy C, Sahn DE. Schooling, marriage, and age at first birth in Madagascar. Popul Stud (Camb). 2015;69(2):219–36. doi: 10.1080/00324728.2015.1053513 . 26217889 10.1080/00324728.2015.1053513

[10] McCleary-Sills J, Hanmer L, Parsons J, Klugman J. Child marriage: a critical barrier to girls' schooling and gender equality in education. The Review of Faith and International Affairs. 2015;13(3):69–80.

[11] Raj A, McDougal L, Silverman JG, Rusch ML. Cross-sectional time series analysis of associations between education and girl child marriage in Bangladesh, India, Nepal and Pakistan, 1991–2011. PLoS One. 2014;9(9):e106210 doi: 10.1371/journal.pone.0106210 ; PubMed Central PMCID: PMCPMC4159189. 25203635 10.1371/journal.pone.0106210 PMC4159189

[12] Lee-Rife S, Malhotra A, Warner A, Glinski AM. What works to prevent child marriage: a review of the evidence. Stud Fam Plann. 2012;43(4):287–303. doi: 10.1111/j.1728-4465.2012.00327.x . 23239248 10.1111/j.1728-4465.2012.00327.x

[13] Omoeva C, Hatch R, Sylla B. Teenage, married, and out of school. Effects of early marriage and childbirth on school dropout. 2014. Available from: http://www.ungei.org/resources/files/EPDC_EarlyMarriage_Report.pdf.

[14] Nguyen MC, Wodon Q. Impact of child marriage on literacy and education attainment in Africa. 2014. Available from: http://allinschool.org/wp-content/uploads/2015/02/OOSC-2014-QW-Child-Marriage-final.pdf.

[15] Field E, Ambrus A. Early marriage, age of menarche, and female schooling attainment in Bangladesh. Journal of Political Economy. 2008;116(5).

[16] Ward AC. The role of causal criteria in causal inferences: Bradford Hill's "aspects of association". Epidemiol Perspect Innov. 2009;6:2 doi: 10.1186/1742-5573-6-2 ; PubMed Central PMCID: PMCPMC2706236. 19534788 10.1186/1742-5573-6-2 PMC2706236

[17] Central Bureau of Statistics (CBS) [Nepal], UNICEF. Nepal Multiple Indicator Cluster Survey 2014. 2015. Available from: http://unicef.org.np/uploads/files/1292493992286457-nepal-mics-kfr-tables-report15-final-27-may-2015.pdf.

[18] Lloyd CB, Mensch BS. Marriage and childbirth as factors in dropping out from school: an analysis of DHS data from sub-Saharan Africa. Popul Stud. 2008;62(1):1–13. doi: 10.1080/00324720701810840 . 18278669 10.1080/00324720701810840

[19] Ghimire A, Samuels F. Change and continuity in social norms and practices around marriage and education in Nepal. 2014. Available from: https://www.odi.org/sites/odi.org.uk/files/odi-assets/publications-opinion-files/9181.pdf.

[20] Nour NM. Child marriage: a silent health and human rights issue. Rev Obstet Gynecol. 2009;2(1):51–6. ; PubMed Central PMCID: PMCPMC2672998. 19399295 PMC2672998

[21] Bajracharya A, Amin S. Poverty, marriage timing, and transitions to adulthood in Nepal. Stud Fam Plann. 2012;43(2):79–92. . 23175948 10.1111/j.1728-4465.2012.00307.x

[22] Nour NM. Health consequences of child marriage in Africa. Emerg Infect Dis. 2006;12(11):1644–9. doi: 10.3201/eid1211.060510 ; PubMed Central PMCID: PMCPMC3372345. 17283612 10.3201/eid1211.060510 PMC3372345

[23] Mensch B, Singh S, Casterline BJ. Trends in the timing of first marriage among men and women in the developing world In: Lloyd BC, Behrman JR, Stromquist PN, Cohen B, editors. The Changing Transitions to Adulthood in Developing Countries: Selected Studies. Washington, DC: National Academies Press; 2005.

[24] Sah N. Does dowry affect age at marriage of women? Some evidence from Terai of Nepal. Nepal Population Journal. 2012;17(16):55–71.

[25] Kalamar AM, Bayer AM, Hindin MJ. Interventions to Prevent Sexually Transmitted Infections, Including HIV, Among Young People in Low- and Middle-Income Countries: A Systematic Review of the Published and Gray Literature. J Adolesc Health. 2016;59(3 Suppl):S22–31. doi: 10.1016/j.jadohealth.2016.05.020 . 27562450 10.1016/j.jadohealth.2016.05.020

[26] The Government of Nepal. National Strategy on Ending Child Marriage 2015 (unpublished). 2016.

[27] Streatfield PK, Kamal N, Ahsan KZ, Nahar Q. Early marriage in bangladesh: not as early as it appears. Asia Population Studies. 2015;11(1):94–110.

