# Supplementary material for: Effect of child marriage on girls' school dropout in Nepal: Analysis of data from the Multiple Indicator Cluster Survey 2014
Source: PLoS One. 2017 Jul 20;12(7):e0180176. doi: 10.1371/journal.pone.0180176 (PMC5519022; doi:10.1371/journal.pone.0180176)
Supplement: S1 Table — (DOCX) [file pone.0180176.s001.docx]

**S1 Table** Associations between child marriage and school participation

|  |  | OR (95% CI) | | Adjusted OR (95% CI)^a^ | |
| --- | --- | --- | --- | --- | --- |
| Married | |  |  |  |  |
|  | No | Reference | | Reference | |
|  | Yes | 0.08 | (0.05 - 0.13)*** | 0.10 | (0.06 - 0.17)*** |
| Age | | 0.45 | (0.37 - 0.56)*** | 0.59 | (0.46 - 0.76)*** |
| Place of residence | |  |  |  |  |
|  | Urban | Reference | | Reference | |
|  | Rural | 0.64 | (0.38 - 1.09) | 1.02 | (0.53 - 1.96) |
| Household wealth status | |  |  |  |  |
|  | Non-poor | Reference | | Reference | |
|  | Poor | 0.84 | (0.59 - 1.21) | 1.01 | ( 0.65 - 1.57) |
| Religion | |  |  |  |  |
|  | Hindu | Reference | | Reference | |
|  | Buddhist | 0.97 | (0.55 - 1.72) | 0.73 | (0.40 - 1.33) |
|  | Muslim | 0.22 | (0.10 - 0.47)*** | 0.15 | (0.06 - 0.38)*** |
|  | Kirat | 0.93 | (0.54 - 1.61) | 1.31 | (0.68 - 2.51) |
|  | Christian | 2.56 | (0.56 - 11.70) | 3.68 | (1.23 - 11.0)* |
|  | Others | 0.09 | (0.01 - 0.83)* | 0.09 | (0.01 - 0.79)* |
| Social classes | |  |  |  |  |
|  | Non-Dalit | Reference | | Reference | |
|  | Dalit | 0.53 | (0.34 - 0.83)** | 0.74 | (0.42 - 1.29) |
| Education of the household head | | |  |  |  |
|  | Secondary or higher | Reference | | Reference | |
|  | Primary | 0.40 | (0.24 - 0.67)** | 0.46 | (0.26 - 0.84)* |
|  | No education | 0.33 | (0.21 - 0.51)*** | 0.41 | (0.25 - 0.66)*** |

n = 1351 for OR; n = 1344 for adjusted OR.

a Analysis adjusted for age, place of residence, household wealth status, religion, social class, and education of the household head.

* Level of significance at *p* < 0.05; ** *p* < 0.01; *** *p* < 0.001
