# Supplementary material for: Effect of child marriage on girls' school dropout in Nepal: Analysis of data from the Multiple Indicator Cluster Survey 2014
Source: PLoS One. 2017 Jul 20;12(7):e0180176. doi: 10.1371/journal.pone.0180176 (PMC5519022; doi:10.1371/journal.pone.0180176)
Supplement: S2 Table — (DOCX) [file pone.0180176.s002.docx]

**S2 Table** Associations between child marriage and school dropout

|  |  | OR (95% CI) | | Adjusted OR (95% CI)^a^ | |
| --- | --- | --- | --- | --- | --- |
| Married | |  |  |  |  |
|  | No | Reference | | Reference | |
|  | Yes | 12.4 | (7.54 - 20.40)*** | 10.04 | (5.84 - 17.25)*** |
| Age | | 2.20 | (1.77 - 2.74)*** | 1.69 | (1.32 - 2.16)*** |
| Place of residence | |  |  |  |  |
|  | Urban | Reference | | Reference | |
|  | Rural | 1.56 | (0.92 - 2.65) | 0.98 | (0.51 - 1.88) |
| Household wealth status | |  |  |  |  |
|  | Non-poor | Reference | | Reference | |
|  | Poor | 1.18 | (0.83 - 1.70) | 0.99 | (0.64 - 1.54) |
| Religion | |  |  |  |  |
|  | Hindu | Reference | | Reference | |
|  | Buddhist | 1.03 | (0.58 - 1.82) | 1.37 | (0.75 - 2.51) |
|  | Muslim | 4.53 | (2.14 - 9.62)*** | 6.83 | (2.6 - 17.92)*** |
|  | Kirat | 1.07 | (0.62 - 1.86) | 0.76 | (0.4 - 1.46) |
|  | Christian | 0.39 | (0.09 - 1.78) | 0.27 | (0.09 - 0.81)* |
|  | Others | 11.65 | (1.21 - 112.48)* | 10.74 | (1.27 - 90.8)* |
| Social classes | |  |  |  |  |
|  | Non-Dalit | Reference | | Reference | |
|  | Dalit | 1.89 | (1.20 - 2.97)** | 1.36 | (0.77 - 2.39) |
| Education of the household head | | |  |  |  |
|  | Secondary or higher | Reference | | Reference | |
|  | Primary | 2.49 | (1.49 - 4.16)** | 2.15 | (1.19 - 3.91)** |
|  | No education | 3.06 | (1.95 - 4.79)*** | 2.46 | (1.51 - 4.02)*** |

n = 1351 for OR; n = 1344 for adjusted OR.

a Analysis adjusted for age, place of residence, household wealth status, religion, social class, and education of the household head.

* Level of significance at *p* < 0.05; ** *p* < 0.01; *** *p* < 0.001
